# Postoperative paralytic ileus following debulking surgery in ovarian cancer patients

**DOI:** 10.3389/fsurg.2022.976497

**Published:** 2022-08-24

**Authors:** Eva K. Egger, Freya Merker, Damian J. Ralser, Milka Marinova, Tim O. Vilz, Hanno Matthaei, Tobias Hilbert, Alexander Mustea

**Affiliations:** ^1^ Department of Gynecology and Gynecological Oncology, University Hospital Bonn, Bonn, Germany; ^2^ Department of Interventional and Diagnostic Radiology, University Hospital Bonn, Bonn, Germany; ^3^ Department of Surgery, University Hospital Bonn, Bonn, Germany; ^4^ Department of Anesthesiology, University Hospital Bonn, Bonn, Germany

**Keywords:** paralytic ileus, ovarian cancer, surgical complexity, complications, anastomotic leakage

## Abstract

**Aim:**

This study aims to evaluate the incidence of postoperative ileus (POI) following cytoreductive surgery in epithelial ovarian cancer (EOC) patients and its impact on anastomotic leakage occurrence and postoperative complications.

**Methods:**

A total of 357 surgeries were performed on 346 ovarian cancer patients between 1/2010 and 12/2020 at our institution. The postoperative course regarding paralytic ileus, anastomotic leakage, and postoperative complications was analyzed by Fisher's exact test and through ordinal logistic regression.

**Results:**

A total of 233 patients (65.3%) returned to normal gastrointestinal functions within 3 days after surgery. A total of 123 patients (34.5%) developed POI. There were 199 anastomoses in 165 patients and 24 leakages (12.1%). Postoperative antibiotics (*p* 0.001), stoma creation (*p* 0.0001), and early start of laxatives (*p* 0.0048) significantly decreased POI, while anastomoses in general (*p* 0.0465) and especially low anastomoses (*p* 0.0143) showed increased POI rates. Intraoperative positive fluid excess >5,000 cc was associated with a higher risk for POI (*p* 0.0063), anastomotic leakage (*p* 0.0254), and severe complications (*p* 0.0012).

**Conclusion:**

Postoperative antibiotics, an early start with laxatives, and stoma creation were associated with reduced POI rates. Patients with anastomoses showed an increased risk for POI. Severe complications, anastomotic leakages, and POI were more common in the case of intraoperative fluid balance exceeding 5,000 cc.

## Introduction

Among gynecologic malignancies, epithelial ovarian cancer (EOC) is the most frequent cause of death in women (1). The most powerful therapeutic tool is optimal cytoreduction to no residual disease, usually requiring high surgical complexity and harboring the risk of increased morbidity (2–6). While enhanced recovery after surgery (ERAS) programs in colorectal and ovarian cancer surgery showed lower morbidity and mortality, no effect was seen on the frequency of postoperative paralytic ileus (POI) (7–10).

The POI relates to severe patient discomfort, including a lack of flatus, abdominal distension, nausea and vomiting, absence of normal bowel sounds, and a delay in the passage of stool, causing prolonged hospital stays, readmissions, reoperations, and possibly anastomotic leakages (11–13). POI represents the most frequent complication following gastrointestinal surgery affecting one out of eight patients (14). Its effect on the quality of life lasts even 3 and 6 months after surgery (15). Due to the lack of a standard POI definition, the I-FEED scoring system was developed by the American society for enhanced recovery after surgery. Here, the general postoperative ileus is split into three groups depending on the severity (measured by 0–2 points) of five different symptoms (oral intake, nauseated feeling, emesis, distension in examination, and duration of symptoms). Each symptom is attributed by 0–2 points according to the severity. A total score of 0–2 points is considered a normal postoperative state. In the case of 3 points or more, a patient is considered to experience POI. In the case of 3–5 points, the POI is milder and defined as gastrointestinal intolerance, and in the case of 6 points, the patients experience a severe form of POI considered as a gastrointestinal dysfunction (16, 17). The relevance of POI regarding the most feared complications as pancreatic fistulas and anastomotic leakages in ovarian cancer patients is unknown (18). Here, we aimed to evaluate the frequency of the postoperative paralytic ileus after cytoreduction in patients with epithelial ovarian cancer and its impact on anastomotic leakage and postoperative morbidity.

## Methods

### Data collection

This study was conducted in accordance with the standards of the ethics committee of the Faculty of Medicine at the University of Bonn, Germany (No.: 14/22). The institutional record database was screened for ovarian cancer patients with cytoreductive surgery between January 2010 and December 2020. Pre-, intra-, and postoperative patient information was recorded from patient's charts, surgery reports, and pathologic findings. All data were evaluated regarding the following postoperative outcomes: postoperative paralytic ileus, anastomotic leakage, and general postoperative complications. In consideration of the I-FEED scoring system, POI was considered in case of an I-FEED score of equal or more than 3 points (17). The peritoneal carcinomatosis index (PCI) as the sum of carcinosis, quantified by size in 13 regions of the abdomen, was retrospectively calculated on the basis of surgery and pathology reports for the evaluation of the tumor load. Depending on the size of the largest tumor nodule in each region, patients received 0 points for no tumor, 1 point for nodules up to 5 mm, 2 points for tumor nodules up to 5 cm, and 3 points for nodules larger than 5 cm. All points were added up to the PCI score in the end (19). The age-adjusted comorbidity index was calculated based on patient's comorbidities that have been weighted analogously to the Charlson comorbidity index generated from patient's charts. Furthermore, one point was added for each decade above 40 years, as age has been identified as a comorbid factor as well (20). The surgical complexity score was evaluated for each patient according to the surgical report. All surgical procedures are attributed by 1–3 points depending on the complexity of the procedure, and the final sum is grouped into low surgical complexity (0–3 points), medium surgical complexity (3–7 points), and high surgical complexity (8–18 points) (21). Using the Memorial Sloan Kettering Cancer Center secondary surgical event score, we classified all postoperative complications from grades 1 to 5 (G1–G5). This score was created for the evaluation of complications within the first 30 days after surgery in cancer patients only. So far, more than 220 different secondary surgical events are included in this score. G1 and G2 complications are minor and treated by bedside care or oral medication and by intravenous medications or transfusions, respectively. G3 complications include anastomotic leakage and are treated by intervention, either radiologic, endoscopic, or operative. G4 complications lead to chronic disability or further organ resection, and G5 complications result in the death of the patient (22). The following aspects of enhanced recovery after surgery are standard of care in all our patients: Placement of a peridural catheter whenever possible, early mobilization on the first day after surgery, and guided mobilization by the nursing staff beginning on the first postoperative day. After mobilization, the urine catheter is removed. Early feeding and drinking should start after 6 h in the case of no anastomosis on the day of surgery, and in the case of an anastomosis, early drinking should start after 6 h on the day of surgery and fluid feeding on the first day after surgery in the morning. There should be immediate removal of the nasogastric tube at the end of surgery. The central venous catheter should be removed latest by the third postoperative day (16, 17).

### Statistical analysis

All variables were analyzed by Fisher's exact test to identify significant correlations of pre-, intra-, and postoperative findings regarding the outcomes of POI, general morbidity, and anastomotic leakage. Differences were considered to be significant at a threshold of ≤0.05. Ordinal logistic regression was used to analyze morbidity in the context of comorbidity and surgical complexity. The test of all slopes equal to zero indicated that complications had a significant association with comorbidity and surgical complexity. The goodness of fit was tested by Pearson’s test and deviance test to verify that the model using link function logit was appropriate. All statistical analyses were performed using Minitab, version 18 (Minitab LLC., State College, PA, USA).

## Results

### General patient characteristics

There were 357 surgeries in a total of 346 patients. Eleven patients received their primary surgery in our department, came back for recurrency, and received surgery again in our department. Fifteen patients received HIPEC (hyperthermic intraperitoneal chemotherapy) at the end of surgery. The median age was 61 years (range: 16–86 years). The physical status classification by the American Society of Anaesthesiologists (ASA) showed 1 ASA-4 patient and 104 ASA-3 patients. The rest of the patients scored ASA-1 and -2. The median PCI was 8 (range: 1–30). The median duration of surgery was 357 min (range: 24–695 min). The median amount of intraoperative kristalloid infusion was 7,500 ml (1,000 ml–21,500 ml). The median positive fluid excess was 6,580 ml (range: 0–19,100 ml). The median number of erythrocyte concentrates was 2 (range: 0–30). The median number of fresh frozen plasma was 0 (range: 0–19). The median highest intraoperative noradrenalin dosing during surgery was 5 µg/ml (range: 0–80 µg/ml). The median number of days in the intensive care unit was 1 day (range: 0–50 days). The median duration of postoperative ventilation was 4 h (range: 0–567 h). The median surgical complexity score was 6 (range: 0–15). Further general patient characteristics are presented in Table 1.

**Table 1:** General patients’ characteristics.

Factor	Number of patients
Primary disease	294 (82.4%)
Recurrent disease	63 (17.6%)
Histology	Serous: 310 (86.8%)
	Endometriod: 24 (6.7%)
	Clear cell: 5 (1.4%)
	Mucinous: 18 (5.0%)
Age-adjusted comorbidity index	0:54 (15.1%)
	1:77 (21.6%)
	2: 91 (25.5%)
	3:68 (19%)
	4:36 (10.1%)
	5:20 (5.6%)
	6:4 (1.1%)
	7:5 (1.4%)
	8:1 (<1%)
	10: 1 (<1%)
Median number of previous surgeries	1 (range: 0–3)
BMI	>40:15 (4.3%)
	>30:58 (16.8%)
	>24:119 (33.52%)
	>19: 155 (44.8%)
	<19:10 (2.9%)
No residual tumor	256 (71.7%)
Residual tumor below 5 mm	40 (11.2%)
Residual tumor below 2 cm	38 (10.6%)
Residual tumor >2 cm	20 (5.6%)
FIGO stages	I: 64 (17.9%)
	II: 20 (5.6%)
	IIIA: 12 (3.4%)
	IIIB: 17 (4.8%)
	IIIC: 207 (58%)
	IVA: 34 (9.5%)
	IVB: 3 (<1%)
Lymphonodectomy	211 (59.1%)
No. of patients with bowel resections	169 (47.3%)
No. of patients with anastomoses	165 (46.2%)
Peridural anesthesia	303 (84.87%)
Patient controlled morphine analgesy	19 (5.3%)
Primary debulking surgery	241 (67.5%)
Interval debulking after chemotherapy	116 (32.5%)

Table 2 presents all 199 anastomoses performed in 165 patients in detail. A total of 130 patients received one anastomosis, 32 patients had two anastomoses, and 3 patients had three anastomoses. A total of 175 involved the large colon and 24 involved only the small bowel. There were 24 leakages (12.06%). Table 2 presents the details.

**Table 2:** 199 Anastomoses.

Anastomosis	Leakage	No leakage
Jejeunojejunostomy	0	6
Ileoileostomy	0	17
Descendorectostomy	18	99
Ileoascendostomy	3	25
Transversotransversostomy	0	3
Transversorectostomy	1	8
Ileorectostomy	1	8
Ascendorectostomy	0	5
Jejunotransversostomy	1	0

### Factors influencing POI

Of all patients, 233 (65.3%) returned to normal gastrointestinal functions within 3 days after surgery. A total of 123 patients (34.5%) were considered as experiencing POI due to a lack of flatus and defecation, nausea, and abdominal distension at least. Fifteen patients needed the placement of a nasogatric tube due to irresistible vomiting. Table 3 presents the day of first defecation after surgery in detail. Table 4 presents the factors associated with a decreased rate of POI and the factors associated with an increased rate of POI.

**Table 3:** First defecation after surgery.

Days after surgery	Number of patients with first postoperative defecation
0 (Day of surgery)	7
1	41
2	68
3	117
4	82
5	27
6	11
7	3

**Table 4:** Factors with increased and decreased POI rates.

Factors associated with a decreased rate of POI	*p*-Value
Postoperative antibiotics	0.001
Stoma creation	0.0001
Start with laxatives on the first postoperative day	0.0048
Factors associated with an increased rate of POI	
Positive fluid excess > 5,000 cc vs. (measured as the sum of intraoperative colloid and crystalloid fluids and subtracted intraoperative urine output)	0.0063
Rectal anastomosis	0.0143
Anastomosis in general	0.0465
Factors without significant influence on POI	
Primary vs. recurrent disease	0.7692
Charlson comorbidity index >8 vs. <8	0.5453
Preoperative laxatives	1.000
No preoperative laxatives	0.7187
Preoperative clysma only	0.1228
Preoperative antibiotics	1.000
PCI > 15	0.8964
Residual tumor	0.8964
Lymphonodectomy	0.3642
Surgical complexity score grading of 3	0.2631
Neoadjuvant chemotherapy	0.0750
Noradrenalin intraoperative >20 µg/ml	0.3019
Peridural catheter	0.1271
Patient-controlled analgesia	0.1271
Kalium >4 mg/L	0.2378

POI, postoperative ileus.

### Postoperative morbidity and duration of hospital stay

There was no significant correlation between morbidity in general (G0 vs. G1–G5) and POI (*p*-value: 0.1634) and between severe postoperative morbidity (G4–G5) and POI (*p*-value: 0.4679). The comorbidity index did not correlate with increasing severe postoperative morbidity (G4–G5; *p*-value: 0.1680). A significant increase in severe postoperative morbidity was seen in the case of increasing surgical complexity (*p*-value: 0.0282).

A positive fluid excess of more than 5,000 ml showed a significant increase in severe complications (G3–G5) (*p*-value: 0.0012), anastomotic leakage (*p*-value: 0.0254), and POI (*p*-value: 0.0063). A significant prolongation of the hospital stay was seen in the case of anastomotic leckage (*p*-value: 0.000), severe postoperative complications (G3–G5) (*p*-value: 0.000), and anastomosis in general (*p*-value: 0.000) but not in the case of a prolonged POI only (>5 days without flatus/defecation, abdominal distension ± vomiting), as seen in Table 5.

**Table 5:** Postoperative morbidity, hospital stay, fluid balance, and anastomotic leakage.

Morbidity	Hospital stay: <17 days	Hospital stay >17 days
G0–3	227	95
G4–G5	6	29
*p*-Value: 0.000		
Anastomotic leakage	Hospital stay: <17 days	Hospital stay > 17 days
Yes	0	24
No	88	53
*p*-Value: 0.000		
Anastomotic leakage	Fluid balance > 5,000 cc	Fluid balance < 5,000 cc
Yes	21	3
No	217	115
*p*-Value: 0.0254		
Morbidity	Fluid balance > 5,000 cc	Fluid balance < 5,000 cc
G0–G2	147	93
G3–G5	91	25
*p*-Value: 0.0012		

Considering surgical complexity, complications, and comorbidity index only, ordinal logistic regression showed significantly more severe postoperative complications (G4–G5) as comorbidity and surgical complexity increased (*p*-value: 0.037). The test of all slopes equal to zero indicated that severe complications had a significant association with comorbidity and surgical complexity. The goodness of fit was tested by Pearson’s test with a *p*-value of 0.249 and deviance test with a *p*-value of 0.243, indicating that the model using link function logit is appropriate. Odds ratios are seen in Table 6.

**Table 6:** Logistic regression table.

Predictor	Odds ratio	95% CI
Comorbidity		
1	2.97	1.05–8.42
2	1.79	0.70–4.54
3	3.23	1.20–8.68
4	1.62	0.52–5.05
5	1.37	0.29–6.37
6	0.15	0.02–1.42
7	1.57	0.12–21.29
Surgical complexity grouped		
2	1.14	0.24–5.39
3	0.69	0.15–3.16

### Interaction between anastomoses and POI

Anastomoses in general and especially low rectal anastomoses led to a significant increase in POI. No correlation was found between POI and anastomotic leakage or POI and small vs. large bowel anastomoses or POI in the case of anastomoses and increasing surgical complexity without stoma creation, as seen in Table 7.

**Table 7:** POI and anastomoses.

Factor	*p*-value
Low rectal anastomoses vs. no anastomosis and POI	**0** **.** **0143**
Small bowel anastomosis vs. no anastomosis and POI	1.000
Anastomosis in general vs. no anastomosis and POI	**0**.**0465**
Anastomotic leakage vs. no leakage and POI	0.3341
Low rectal anastomosis and leakage vs. no leakage and POI	0.1589
Anastomotic leakage vs. no leakage	0.3341
Large bowel anastomosis vs. small bowel anastomosis only	0.3341
Anastomotic leakage considering only patients without stoma (day 4)	0.5405
Increasing surgical complexity in patients with rectal anastomoses and without stoma creation	1.000

POI, postoperative ileus.

## Discussion

There is a broad consensus that intestinal manipulation leads to intestinal inflammation causing impaired gastrointestinal motility (12, 23, 24). Advances in laparoscopic techniques have significantly reduced the immunological response and POI due to less tissue trauma and less stimulation of the bowel, vessels, and nervous system (25, 26). As ovarian cancer surgery remains an open surgical procedure, we could not show an association between POI and increasing surgical complexity. Like others, we saw increasing morbidity as surgical complexity increased (20). This matches well with data from colonic cancer, showing that the open approach in right-sided colonic resections only increased surgical complexity and significantly increased POI compared to the laparoscopic approach in colonic cancer (25, 26). In colorectal surgery, POI is considered a promoter of postoperative complications (15). In our cohort, we did not observe any association between POI and postoperative complications. Consistent with the recent literature in ovarian cancer surgery, a significant increase in severe complications apart from POI was found with increasing surgical complexity (21). While comorbidity only had no influence on POI or complication rate, the combination of increasing comorbidity and increasing surgical complexity showed a significant increase in severe postoperative complications.

Although there is strong evidence for the use of preoperative antibiotics in colorectal surgery to prevent POI, surgical site infections, and anastomotic leckage, there is little evidence for the same outcomes for the use of postoperative i.v. antibiotics (27, 28). Postoperative antibiotic use for 3–5 days resulted in a significant increase in the proportion of patients with defecation within the first 3 days in our cohort. Furthermore, we saw a significant decrease in POI in the case of laxative use starting on the first postoperative day. Here, metoclopramide and bisacodyl were used as a standard in our clinic for the first 3 days. Nevertheless, there is only weak evidence for using laxatives in colorectal surgery to prevent POI (29). Caffeine intake and coffee as a beverage, on the other hand, have shown promising results regarding prevention of POI and safety after colorectal surgery (30–32).

Fluid overload in colorectal surgery has been identified as a promoter of POI and increased complications such as anastomotic leakage. Especially crystalloid fluids are suspected of producing splanchnic edema and thus escalating abdominal pressure, which in turn reduces mesenteric blood flow and contributes to tissue hypoxemia and impaired anastomotic healing (33–36). Fluid management in ovarian cancer patients is often challenging due to intraoperative fluid shifts from the endovascular space to the intra-abdominal and interstitial space in part due to an increased expression of vascular leakage genes; this leads to increasing amounts of crystalloid infusion intraoperatively and slower bowel function recovery (37, 38). In our cohort, we saw a significant increase in POI, anastomotic leakage, and severe complications at a fluid excess of 5,000 cc and more. Compared to colorectal surgery, less data are available for ovarian cancer surgery regarding limited fluid administration, but it seems safe and decreases the time of gastrointestinal impairment and hospital stay (36). While postoperative ileus is not only a matter of gastrointestinal resections, we identified anastomoses, especially low anastomoses, which are the most common anastomoses in the case of ovarian cancer, as a significant additional risk factor for POI (39).

Diverting ileostomies may reduce the clinical severity of anastomotic leakage, but no reduction of leakages is seen in the case of rectal cancer, remaining a controversial topic (40). Especially high output diverting ileostomies are suspected to be at risk for POI, readmission, and subsequent ileus (40). In ovarian cancer, anastomoses are generally 3 cm–5 cm higher than in rectal cancer. Here, a preventive effect of diverting ileostomies on anastomotic leakage was seen (41). A recent study found more anastomotic leakage in the case of medium-low colorectal anastomosis <10 cm from the anal verge (42). Furthermore, in our actual study, we found a significant preventive effect for POI. Nevertheless, this may not be turned into a recommendation for stoma creation in the case of colorectal anastomoses as anastomotic leakage rates are generally low and POI will be overcome without stoma creation (43). Further improvements in surgical techniques such as total mesorectal resections with preservation of the superior rectal artery and improvements in the presurgical nutritional status may even lower the rate of colorectal anastomotic leakage (44, 45).

Most likely due to a different policy between the USA and Germany regarding the duration of in-hospital length of stay after major abdominal surgeries, we saw no significant association between POI and prolonged in-hospital length of stay (13).

Our study has some limitations. Due to the retrospective evaluation of POI, a clear distinction between gastrointestinal intolerance and gastrointestinal dysfunction was not possible, although it may be important, especially with regard to POI-associated morbidity. Therefore, over- and underestimation of the true postoperative gastrointestinal impairment may be possible.

Additionally, the fluid status depends on a number of factors such as the preoperative fluid status, preoperative bowel preparation, duration of surgery, blood loss, and patient fluid output by urine and intra-abdominally with respect to the amount of resected tumor. Therefore, more accurate measurements are needed in further studies to further define fluid overload in ovarian cancer patients.

The strength of our study is the homogenous patient population, of only patients undergoing cytoreduction for epithelial ovarian cancer. Furthermore, we are the first to show that increasing surgical complexity does relate to increased morbidity but does not affect POI.

## Conclusion

The postoperative paralytic ileus may be reduced in the case of postoperative antibiotic use, early initiation of laxatives, and stoma creation. While antibiotics and laxatives are easy to implement, no recommendation for stoma creation can be drawn from our data due to the negative side effects of a stoma and POI will be overcome without a stoma. Optimal fluid management in ovarian cancer patients continues to be challenging as postoperative complications, postoperative paralytic ileus, and anastomotic leakage increase considerably with an increase in fluid excess. Duration of hospitalization is prolonged in the case of anastomotic leakage and severe morbidity but not in the case of a postoperative ileus.

## Data Availability

The raw data supporting the conclusions of this article will be made available by the authors, without undue reservation.
